# Glutamatergic synaptic deficits in the prefrontal cortex of the Ts65Dn mouse model for Down syndrome

**DOI:** 10.3389/fnins.2023.1171797

**Published:** 2023-09-28

**Authors:** Aurore Thomazeau, Olivier Lassalle, Olivier J. Manzoni

**Affiliations:** ^1^Côte d’Azur, CNRS UMR7275, Institut de Pharmacologie Moléculaire et Cellulaire, Valbonne, France; ^2^Université Aix-Marseille, Marseille, France; ^3^INMED, INSERM U1249, Marseille, France

**Keywords:** intellectual disability, Down syndrome, Ts65Dn, excitatory synapse, long-term synaptic plasticity, prefrontal cortex, pyramidal neuron

## Abstract

Down syndrome (DS), the most prevalent cause of intellectual disability, stems from a chromosomal anomaly resulting in an entire or partial extra copy of chromosome 21. This leads to intellectual disability and a range of associated symptoms. While there has been considerable research focused on the Ts65Dn mouse model of DS, particularly in the context of the hippocampus, the synaptic underpinnings of prefrontal cortex (PFC) dysfunction in DS, including deficits in working memory, remain largely uncharted territory. In a previous study featuring mBACtgDyrk1a mice, which manifest overexpression of the *Dyrk1a* gene, a known candidate gene linked to intellectual disability and microcephaly in DS, we documented adverse effects on spine density, alterations in the molecular composition of synapses, and the presence of synaptic plasticity deficits within the PFC. The current study aimed to enrich our understanding of the roles of different genes in DS by studying Ts65Dn mice, which overexpress several genes including *Dyrk1a*, to compare with our previous work on mBACtgDyrk1a mice. Through *ex-vivo* electrophysiological experiments, including patch-clamp and extracellular field potential recordings, we identified alterations in the intrinsic properties of PFC layer V/VI pyramidal neurons in Ts65Dn male mice. Additionally, we observed changes in the synaptic plasticity range. Notably, long-term depression was absent in Ts65Dn mice, while synaptic or pharmacological long-term potentiation remained fully expressed in these mice. These findings provide valuable insights into the intricate synaptic mechanisms contributing to PFC dysfunction in DS, shedding light on potential therapeutic avenues for addressing the neurocognitive symptoms associated with this condition.

## 1. Introduction

Down syndrome (DS), the most common form of intellectual disability, is a chromosomal disorder caused by having all or part of an extra chromosome 21. This additional copy introduces ~300 extra genes, resulting in a wide range of significant clinical phenotypes. The shared feature among all individuals with DS is intellectual disability, which manifests as specific deficits in learning and memory (Chapman and Hesketh, 2000). The underlying neural mechanisms of these alterations remain poorly understood, but potential factors could include defects in neural network formation, information processing, and brain plasticity.

Mouse models of neurological disorders are invaluable tools for identifying the cellular mechanisms at play in these conditions. Several animal models mimicking DS alterations have been developed, with the most extensively studied being the Ts65Dn mouse. This mouse model has a segmental trisomy of mouse chromosome 16, bearing three copies of genes equivalent to those on human chromosome 21 (Hsa21), many of which are conserved between mice and humans (Davisson et al., 1990; Gardiner et al., 2003; Sturgeon and Gardiner, 2011; Rueda et al., 2012). This trisomic region includes ~140 genes and largely overlaps with the Hsa21 region believed to be responsible for many DS phenotypes, including intellectual disability (Korenberg et al., 1994). Amongst these triplicated genes, the *Dyrk1a* gene has been proposed as a major contributor to the intellectual disability and microcephaly in DS (Courcet et al., 2012). Ts65Dn mice exhibit DS-relevant behavioral, cellular, and molecular phenotypes, such as working memory alterations, long-term memory deficits, hyperactivity, disrupted neurogenesis, and synaptic plasticity, and a general over-inhibition (Holtzman et al., 1996; Gardiner and Davisson, 2000; Belichenko et al., 2004, 2007; Kleschevnikov et al., 2004; Rueda et al., 2012; Ruiz-Mejias, 2019). Much of the research on synaptic plasticity has focused on the hippocampus, a brain structure vital for learning and memory (Cramer and Galdzicki, 2012).

The prefrontal cortex (PFC) is acknowledged as a key brain region controlling executive cognitive processes like planning, cognitive flexibility, working memory, and emotional behavior (Goldman-Rakic, 1990; Seamans et al., 1995). Dysfunctions in the PFC are common in numerous neuropsychiatric diseases, including DS (Goto et al., 2010). Neuropsychological assessments of individuals with DS have highlighted deficits in executive functions (Lanfranchi et al., 2010). The specific role of the triplication of these ~140 genes in PFC malfunction and the synaptic basis for the working memory impairments seen in DS individuals is not yet fully understood (Pennington et al., 2003; Lanfranchi et al., 2010).

In this study, we analyzed the intrinsic characteristics and glutamatergic synaptic plasticity of deep-layer pyramidal neurons in the PFC of adult male Ts65Dn mice. Our research focus was directed toward the synapses in the deep layers of the prefrontal cortex (PrPFC), specifically from layer II-III to V-VI. We targeted the deep-layer pyramidal neurons situated in layer V-VI, which serve as a central source of output from the prefrontal cortex. Notably, the downward projections from layers II/III to layers V/VI play a pivotal role in information processing, with the pyramidal cells in layer V projecting to subcortical regions.

We focused solely on male Ts65Dn mice as significant over-expression of the DS candidate gene *Dyrk1a* in the cerebral cortex has been detected in males, but not females (Hawley et al., 2022). Our findings revealed alterations in the intrinsic properties of pyramidal neurons and specific changes in synaptic plasticity. These results uncover functional abnormalities in the PFC of male Ts65Dn mice, which may contribute to the cognitive and behavioral impairments observed in individuals with DS.

## 2. Materials and methods

### 2.1. Animals and ethics statement

All animal experiments were performed according to the criteria of the European Communities Council Directive (86/609/EEC). The B6EiC3Sn.BLiA-Ts(1716)65Dn/DnJ [known as Ts65Dn] mice were obtained from Dr. Jean Delabar’s laboratory. Ts65Dn mice (Davisson et al., 1993) were maintained on a B6/C3H background and genotyped as described previously (Reinholdt et al., 2011). All mice were weaned at 21 days. After weaning, they were caged socially in same-sex groups. Male Ts65Dn mice and wild type littermate controls were used at 4 to 6 months of age.

### 2.2. Acute prefrontal cortex slice preparation

PFC slices were prepared as described (Lafourcade et al., 2011). Mice were anesthetized with isoflurane and decapitated. The brain was sliced (300 μm) in the coronal plane (Integraslice, Campden Instruments, Leicester, U.K.) and maintained in physiological saline (4°C). Slices were stored for 30 min at 32–35°C in artificial cerebrospinal fluid (ACSF) containing 126 mM NaCl, 2.5 mM KCl, 2.4 mM MgCl2, 1.2 mM CaCl2, 18 mM NaHCO3, 1.2 mM NaH2PO4 and 11 mM glucose, equilibrated with 95% O2/5% CO2. Slices were stored at 22 ± 2°C until recording.

### 2.3. Electrophysiology

Whole-cell patch-clamp and field excitatory postsynaptic potential (fEPSP) were recorded from layer V/VI pyramidal cells in coronal slices of mouse prelimbic PFC (Lafourcade et al., 2011; Thomazeau et al., 2014). For recording, slices were superfused (2 ml/min) with ACSF at 32–35°C. Picrotoxin (100 μM) was added to block GABA-A receptors. To evoke synaptic currents, 150–200 μs stimuli were delivered at 0.1 Hz through a glass electrode placed in layer II/III.

**Figure 1:**
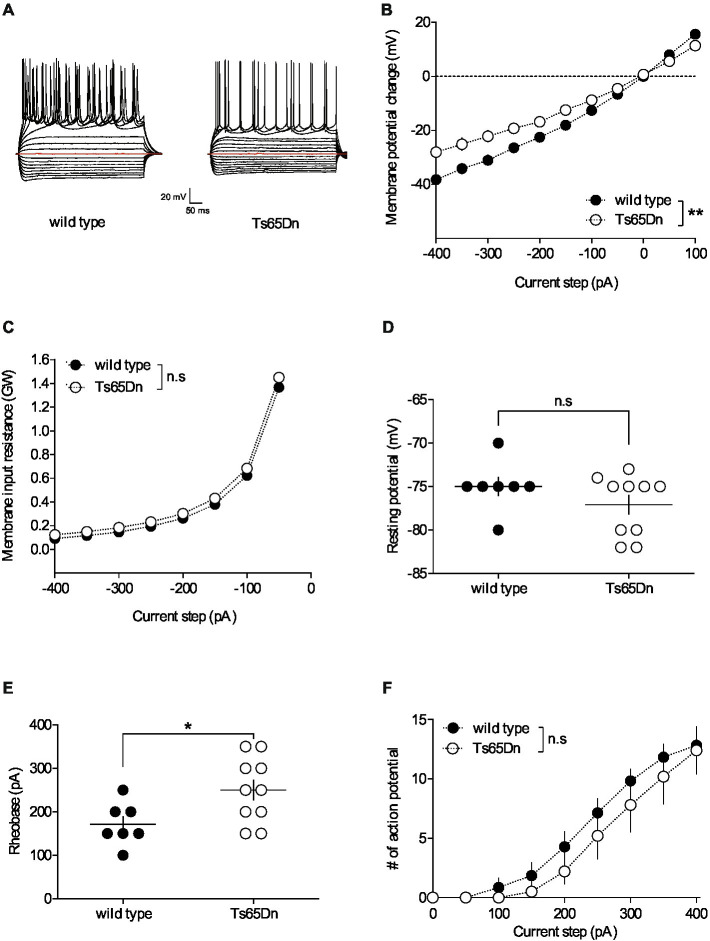
The intrinsic properties of pyramidal neurons in layers V/VI of the prefrontal cortex are altered in Ts65Dn mice. PFC pyramidal neurons were patched in current-clamp mode and current was injected. The stimulation protocol consisted in 19 steps of current, 500 ms-long, with increment of 50 pA, starting at −400 pA. **(A)** Typical membrane responses to somatic current steps of PFC pyramidal neurons from wild-type and Ts65Dn mice. **(B)** Summary of current-voltage (I-V) curves recorded in pyramidal neurons of both strains showing an increase in inward rectification in Ts65Dn mice (white symbols; *n* = 10 neurons from 4 mice) compared to wild-type mice [black symbols; *n* = 7 neurons from 5 mice; two-way repeated measures ANOVA, genotype: *F*_(1,15)_ = 9.932, **p = 0.0066]. **(C)** The membrane input resistance was similar in wild-type and Ts65Dn mice (two-way repeated measures ANOVA, genotype: *F*_(1,15)_ = 0.6498, n.s. *p* = 0.4328). Resting membrane potential is similar in both genotypes (**D.** −75.00 ± 1.09 mV, *n* = 7 neurons from 5 wild-type mice, black symbols; −77.10 ± 1.10 mV, *n* = 10 neurons from 4 Ts65Dn mice, white symbols; n.s. *p* > 0.05, Mann-Whitney test). In contrast the rheobase, i.e. the minimal current necessary to evoke action potential firing, was higher in Ts65Dn mice (**E.** 171.40 ± 18.44 pA, *n* = 7 neurons from 5 mice wild-type mice, black symbols; 250.00 ± 23.57 pA, *n* = 10 neurons from 4 Ts65Dn mice, white symbols; *p = 0.0438, Mann-Whitney test). The horizontal line represents the mean value. (**F.** The summary of current-discharge curves indicates that the number of action potentials elicited in response to current injection steps is similar in pyramidal neurons of Ts65Dn mice (*n* = 10 neurons from 4 mice, white symbols) and wild type mice (*n* = 7 neurons from 5 mice, black symbols; two-way repeated measures ANOVA, genotype: *F*_(1,15)_ = 0.697, n.s. *p* = 0.4169). Error bars represent standard error to the mean.

**Figure 2:**
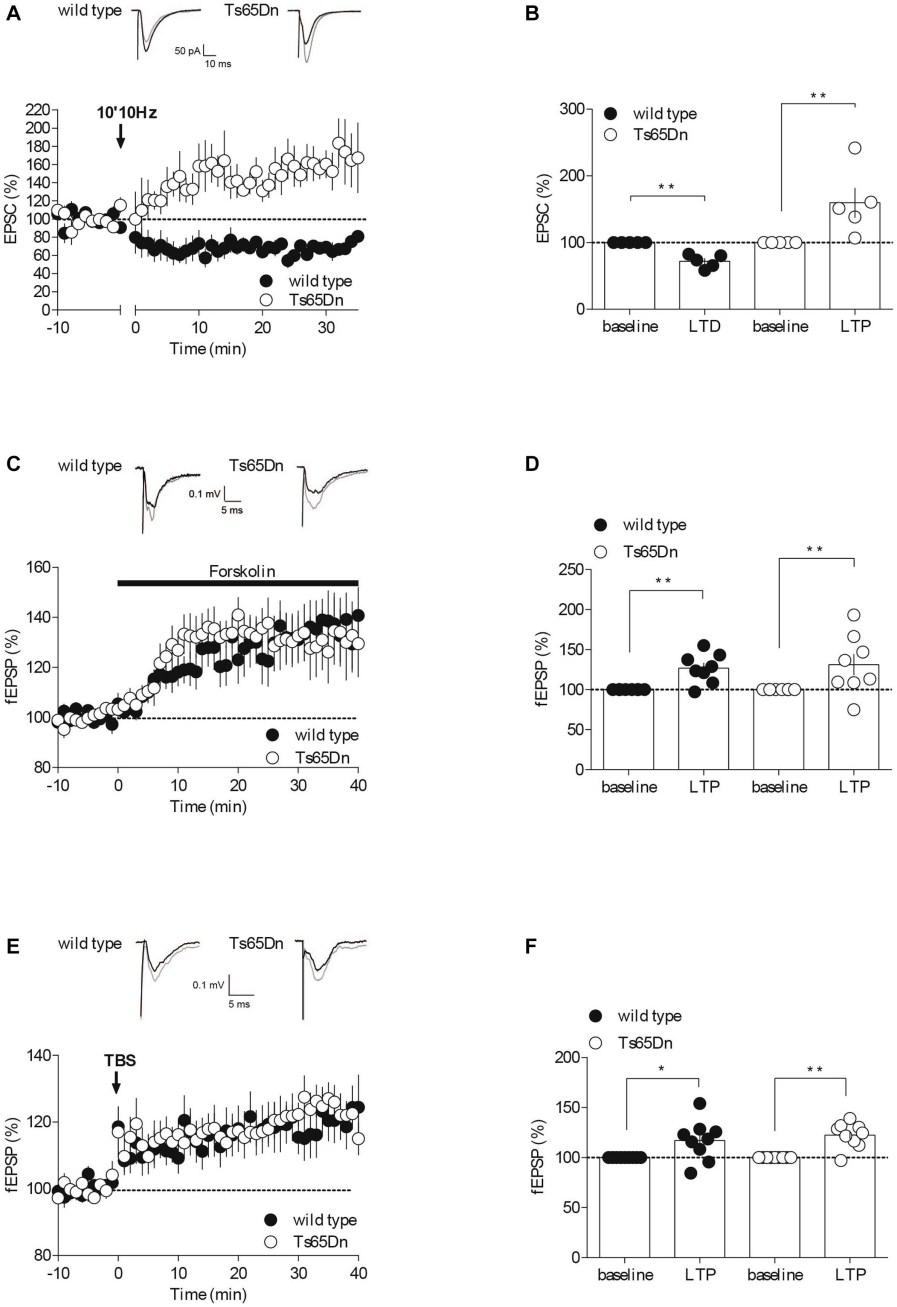
Synaptic plasticity is partially affected in Ts65Dn mice. Endocannabinoid-dependent LTD is absent in Ts65Dn mice. **(A)** Low frequency stimulation of layers II/III induces the long-term depression of the amplitude of excitatory post-synaptic currents (EPSCs) recorded in pyramidal neurons from layers V/VI of the PFC in wild-type mice. In Ts65Dn mice, this protocol triggered instead a long-term potentiation in the amplitude of EPSCs recorded in pyramidal neurons from layers V/VI of the PFC. Representative traces averaged from EPSC response before (black) and 30 min after plasticity induction (gray) in mice from either genotype. Average time courses of mean EPSC amplitude were normalized to baseline. Error bars represent standard error to the mean. **(B)** Average EPSC as percentage of the baseline recapitulating the data (wild-type: *n* = 5 neurons from 5 mice, black symbols, 72.14 ± 4.53, 30–35 min versus 100% baseline; **p = 0.0079, Mann–Whitney test; Ts65Dn mice: *n* = 5 neurons from 5 mice, white symbols, 159.83 ± 22.42, 30–35 min versus 100% baseline; **p = 0.0079, Mann–Whitney test). Error bars represent standard error to the mean. The AC/cAMP-dependent facilitation is intact in Ts65Dn mice. **(C)** Incubation with the adenylate cyclase activator, forskolin, in the perfusion bath leads to an increase in the amplitude of the field excitatory postsynaptic potentials (fEPSPs) recorded in layers V/VI in wild-type mice and in Ts65Dn mice. Representative traces averaged from fEPSP response before (black) and 35 min after plasticity induction (gray) in mice from either genotype. Average time courses of mean fEPSP amplitude were normalized to baseline. Error bars represent standard error to the mean. **(D)** Average fEPSP as percentage of the baseline recapitulating the data (wild-type: *n* = 8 slices from 8 mice, black symbols, 126.82 ± 6.62%, 35–40 min versus 100% baseline; **p = 0.0104, Mann–Whitney test; Ts65Dn mice: *n* = 8 slices from 8 mice, white symbols, 131.2 ± 13.26%, 35–40 min versus 100% baseline; **p = 0.0057, Mann–Whitney test). Error bars represent standard error to the mean. Long-term potentiation dependent induced by TBS is preserved in the Ts65Dn mouse model. **(E)** A theta burst stimulation of PFC layers II/III induces long-term potentiation of the amplitude of the field excitatory postsynaptic potentials (fEPSPs) recorded in layers V/VI in wild-type mice and in Ts65Dn. Representative traces averaged from fEPSP response before (black) and 35 min after plasticity induction (gray) in mice from either genotype. Average time courses of mean fEPSP amplitude were normalized to baseline. Error bars represent standard error to the mean. **(F)** Average fEPSP as percentage of the baseline recapitulating the data (wild-type: *n* = 9 slices from 9 mice, black symbols, 117.07 ± 6.69, 35–40 min versus 100% baseline; *p = 0.0361, Mann–Whitney test; Ts65Dn mice: *n* = 9 slices from 9 mice, 122.43 ± 4.16, 35–40 min versus 100% baseline; **p = 0.0019, Mann–Whitney test). Error bars represent standard error to the mean.

For whole-cell patch-clamp experiments, pyramidal neurons were visualized using an infrared microscope (BX-50, Olympus). Experiments were performed with electrodes containing 128 mM potassium gluconate (KGlu), 20 mM NaCl, 1 mM MgCl2, 1 mM EGTA, 0.3 mM CaCl2, 2 mM Na2 + -ATP, 0.3 mM Na + -GTP, 10 mM glucose buffered with 10 mM HEPES, pH 7.3, osmolarity 290 mOsm. Electrode resistance was 4–6 MOhm. If access resistance (no compensation, <25 MOhm) changed by >20%, the experiment was rejected (Thomazeau et al., 2014). Neuronal intrinsic excitability was assessed during a current clamp protocol. Membrane voltage response, resting membrane potential, input resistance, rheobase and the number of action potentials were determined by applying current steps ranging from −400 to +400 pA in increments of +50 pA, each lasting 500 ms. The membrane voltage response was evaluated based on the steady-state voltage during hyperpolarizing and depolarizing current injections (Figure 1B). Input resistance was computed as the change in membrane voltage (∆mV) divided by the injected current (pA) (Figure 1C). Resting membrane potential was assessed at the beginning of the whole-cell recording during the current clamp protocol (Figure 1D). Rheobase was defined as the minimum current necessary to elicit an action potential (Figure 1E). The number of action potentials was determined for each depolarizing current step lasting 500 ms (Figure 1F). For voltage-clamp experiments (Figures 2A,B), evoked EPSCs were recorded at −70 mV and LTD was induced by 10 min stimulation at 10 Hz (Lafourcade et al., 2011).

For fEPSP experiments (Figures 2C–F), input–output profiles were recorded for all fEPSP recordings. In time course experiments, the stimulation intensity was that necessary give a response 40–60% of the maximal. fEPSPs were recorded at 0.1 Hz. The presynaptic adenylate cyclase (AC)/cAMP signaling pathway-dependent facilitation was induced by bath application of the AC activator forskolin at 10 μM. NMDAR-LTP was induced using a theta-burst stimulation (TBS) protocol consisting of five trains of burst with four pulses at 100 Hz, at 200 ms interval, repeated four times at intervals of 10 s (Thomazeau et al., 2014). The glutamatergic nature of the fEPSP was confirmed at the end of the experiments using the glutamate receptor antagonist 6,7-dinitroquinoxaline-2, 3-dione (DNQX, 20 μM), that blocked the synaptic component without altering the non-synaptic component (not shown).

### 2.4. Data acquisition and analysis

Due to the strong phenotypes, the experimenters were not blind to the genotypes, but no data were excluded before statistical analysis (GraphPad Software Inc., La Jolla, CA). Data were recorded on a MultiClamp700B, filtered at 2 kHz, digitized (20 kHz, DigiData 1440A), collected using Clampex 10.2 and analyzed using Clampfit 10.2 (all from Molecular Device, Sunnyvale, USA). Both amplitude and area of fEPSPs and EPSCs were computed, and only amplitude was display, the area giving the same results. The magnitude of LTD and LTP was calculated, respectively, 30–35 min and 35–40 min after induction as percentage of baseline responses. To perform current–voltage (I–V) curves and to test the excitability of pyramidal neurons, a series of 500 ms long hyperpolarizing and depolarizing current steps, from −400 to +400 pA with 50 pA increment, were applied immediately after breaking in the cell. The rheobase has been determined as the minimum current intensity required to induce a first action potential.

### 2.5. Drugs

Drugs were added at the final concentration to the ACSF. Picrotoxin was from Sigma (St. Quentin Fallavier, France). DNQX was from the National Institute of Mental Health’s Chemical Synthesis and Drug Supply Program (Rockville, MD, USA). Forskolin was from Tocris (Bristol, UK).

### 2.6. Statistical analysis

The value n corresponds to the number of individual cells or slice per animal. All values are given as mean ± standard error to the mean (s.e.m). Statistical analysis was performed with Prism 7.0 (GraphPad Software Inc., La Jolla, CA). Two sample comparisons were made with the Mann–Whitney *t-*test. Multiple comparisons (genotype and current injection) were analyzed using repeated measure two-way ANOVA with *post hoc* Sidak’s test. Statistical significance was set at *p* < 0.05.

## 3. Results

### 3.1. Intrinsic properties of PFC pyramidal neurons are changed in Ts65Dn mice

Neuronal pathways are plastic, continually adapting in response to stimuli. These alterations can occur at synaptic locations (areas where neurons connect) as well as non-synaptic locations throughout the neuron. Non-synaptic, or intrinsic, plasticity can be defined as a change in the neuron’s inherent excitability that is independent of changes in synaptic transmission. To understand the impact of the segmental trisomy 16 of Ts65Dn mice on neuronal membrane properties, the basic intrinsic properties of prefrontal cortex (PFC) pyramidal neurons were first examined. Pyramidal neurons from layers V/VI of the PFC were recorded in the “current-clamp” mode to assess their membrane profile in response to a range of somatic current steps, from −400 to +400 pA in 50 pA increments (Figure 1A). The average curves illustrating the relationship between the current injected into the neuron and the resulting change in membrane potential (I/V curves) revealed differences between Ts65Dn mice and their wild-type euploid littermates. Notably, the pyramidal neurons of the aneuploid mice exhibited increased inward rectification, evidenced by a differential deviation from linearity in the I/V plots (Figure 1B). However, there was no change detected in the membrane input resistance (Figure 1C) and the membrane potential (Figure 1D). The rheobase, or the minimum current intensity needed to trigger the first action potential, was higher in Ts65Dn mice, indicating a decrease in membrane excitability (Figure 1E). Lastly, the number of action potentials emitted following incremental current injection was found to be similar between the two mouse strains (Figure 1F).

### 3.2. PFC synaptic plasticity in the Ts65Dn mice

The intrinsic properties of neurons, which dictate their spiking patterns and level of excitability, are critical for their computational capabilities. Changes in these non-synaptic properties, as well as in synaptic properties, can influence synaptic efficacy. Long-term modifications of synaptic efficacy, referred to as synaptic plasticity, are believed to play crucial roles in cognition, learning, and the formation of memory. Following our assessment of neuronal intrinsic properties, we next evaluated synaptic plasticity in the prefrontal cortex of Ts65Dn mice. We compared opposite forms of synaptic plasticity that rely on distinct mechanisms in our mouse groups. These forms included endocannabinoid-mediated long-term depression (eCB-LTD), presynaptic adenylyl cyclase/cyclic adenosine monophosphate signaling pathway-dependent facilitation (AC/cAMP-facilitation), and N-methyl-D-aspartate receptor-dependent long-term potentiation (NMDAR-LTP) (Figure 2).

#### 3.2.1. Induction of endocannabinoid-dependent long-term depression appears to fail in Ts65Dn mice

The endocannabinoid (eCB) system plays a significant role in synaptic plasticity, and it might contribute to the development of mood disorders associated with prefrontal cortex (PFC) dysfunction (Hill et al., 2009; Scheyer et al., 2017). In response to neuronal activity, eCBs are released by the postsynaptic compartments, and they retrogradely activate presynaptic cannabinoid receptors, resulting in long-term depression (LTD) by reducing neurotransmitter release (Robbe et al., 2002; Puente et al., 2011; Araque et al., 2017). To assess whether eCB-LTD was impaired in the PFC of Ts65Dn mice, we recorded excitatory postsynaptic currents (EPSCs) isolated with Picrotoxin (100 μM) in layer V/VI pyramidal neurons patch-clamped at –70 mV. We then gauged the impact of a low-frequency tetanic stimulus protocol (10 min at 10 Hz). Consistent with our previous studies (Lafourcade et al., 2007, 2011; Thomazeau et al., 2014), low-frequency tetanic stimulation in the prefrontal layers II/III triggered a lasting decrease in the efficacy of excitatory synapses, as measured by the amplitude of EPSCs, onto PFC layer V/VI pyramidal neurons in wild-type mice. In stark contrast, the identical protocol led to an increase in the EPSCs recorded in the prefrontal layer V/VI pyramidal neurons of Ts65Dn mice (Figures 2A,B).

#### 3.2.2. Protein kinase A-mediated long-term potentiation occurs to remain unaltered in Ts65Dn mice

To determine if the release probability was generally affected, we next investigated a presynaptic form of LTP, specifically Adenylyl cyclase (AC)/cAMP-facilitation. Adenylyl cyclase is an enzyme that transforms ATP into cAMP, which subsequently activates protein kinase A, thereby facilitating the release of the transmitter (Chavez-Noriega and Stevens, 1994; Weisskopf et al., 1994). We used the AC activator Forskolin in a bath application and observed its effect on the amplitude of field excitatory postsynaptic potentials (fEPSPs) recorded in PFC layers V-VI following electrical stimulation of layer II-III. The synaptic enhancement induced by the pharmacological activation of AC with Forskolin appeared to be typical in the aneuploid strain (Figures 2C,D), indicating that this form of plasticity seems to be preserved in Ts65Dn animals. The synaptic enhancement resulting from the pharmacological activation of AC using Forskolin seemed to exhibit a typical response in the aneuploid strain (Figures 2C,D). This suggests that this specific form of plasticity is conserved in Ts65Dn animals.

#### 3.2.3. Theta burst stimulation-induced long-term potentiation is unaffected in Ts65Dn mice

NMDAR-dependent long-term potentiation (LTP) is arguably the most widely expressed and comprehensively studied form of synaptic potentiation (Volianskis et al., 2015). We evaluated this postsynaptic form of LTP at the PFC synapses from layers II-III to V-VI in both wild-type and Ts65Dn mice by recording evoked fEPSPs and examining the impact of a theta burst stimulation (TBS) protocol (five burst trains of four pulses at 100 Hz, 200 ms apart, repeated four times at 10 s intervals). The LTP was not affected in Ts65Dn mice (Figures 2E,F). Indeed, TBS of PFC layers II/III induced a comparable increase in the measured amplitude of the evoked field potential response in V/VI layers in both mouse strains.

## 4. Discussion

In this study, we found altered intrinsic properties and some impaired synaptic plasticity in the PFC of Ts65Dn mice, a model of DS with overexpression of around 130 genes from the mouse analog of Hsa21, Mmu16, including Dyrk1a. Notably, our results contrast with those reported in mBACtgDyrk1a mice.

### 4.1. Intrinsic neuronal properties

Whole-cell patch clamp recordings allowed investigating PFC pyramidal neurons’ cellular intrinsic properties to detect alterations in neuronal excitability. In an earlier study, we showed that the intrinsic properties of PFC layer V/VI pyramidal neurons were not affected in mBACtgDyrk1a mice (Thomazeau et al., 2014). In marked contrast, we report here that Ts65Dn mice exhibit an augmented inward rectification and a higher rheobase (Figure 1), reflecting a decrease in neuronal excitability. This is in line with the decreased neuronal excitability already reported in somatosensory cortex layer IV neurons and in dissociated neuronal cultures from the hippocampi of Ts65Dn mice (Cramer et al., 2015; Stern et al., 2015). This reduced intrinsic excitability has been linked to an increase expression level of the inwardly rectifying potassium channel Kir3.2, encoded by the *Kcnj6* gene, one of the triplicated genes in the Ts65Dn mice (Harashima et al., 2006; Best et al., 2007, 2012). Strategies to pharmacologically reduce this channel function could be explored to normalize the intrinsic properties of PFC pyramidal neurons in Ts65Dn mice.

### 4.2. Synaptic plasticity range

Changes in intrinsic, i.e., non-synaptic, properties of neurons can impact their ability to integrate synaptic information. To assess whether dynamic changes at the layers II-III to V-VI pyramidal cell synapses level of pyramidal neurons from PFC were affected in Ts65Dn mice, we then examined several forms of synaptic plasticity that rely on distinct pre- and postsynaptic mechanisms. Interestingly, eCB-LTD appears to be affected in both mouse models (Figures 2A,B; Thomazeau et al., 2014). In contrasts, TBS induced-LTP and AC/cAMP-facilitation was solely disrupted in the mBACtgDyr1a mice (Figures 2C–F; Thomazeau et al., 2014, unpublished data). Thus, glutamatergic synaptic plasticity appears to be much less altered in Ts65Dn mice compared to mBACtgDyrk1a mice. Further investigations should be conducted to assess basic synaptic properties in Ts65Dn PFC synapses, in order to understand whether changes in synaptic properties, such as PFC synaptic excitability, would explain the alteration in synaptic plasticity observed in Ts65Dn mice, i.e., by a change in the number of active excitatory synapses recruited during plasticity induction. Basic synaptic properties could be profiled by (i) establishing as input/output relationships to evaluate synaptic efficacy; (ii) quantifying the progressive inhibition of NMDAR-mediated EPSCs by an open-channel blocker of NMDAR (Chavis and Westbrook, 2001; Thomazeau et al., 2014) or measuring paired-pulse ratio -a form of short-term synaptic plasticity that depends on release probability- to evaluate glutamate release probability; (iii) measuring excitation-spike coupling (Daoudal et al., 2002; Thomazeau et al., 2014) to assess how excitatory synaptic inputs are integrated into action potential; and (iv) by identifying NMDAR subunit composition of PFC pyramidal synapses in Ts65Dn mice. Moreover, TBS induced-LTP appears to be intact, but it would be crucial to pharmacologically verify that its induction is mediated by activation of NMDARs. Out of all the plasticity tested, only eCB-LTD seems to be impaired. Mechanistically, this form of plasticity is more complex than NMDAR- or mGluR-dependent plasticity, as it involves a series of pre- and postsynaptic loci and processes. This could explain why it is more sensitive to genetic modifications. Surprisingly, the eCB-LTD protocol, lead to LTP in Ts65Dn mice.

Additional experiments are required to unravel the mechanisms facilitating the induction of LTP rather than LTD in response to low-frequency tetanic stimulation in Ts65Dn mice. The most straightforward explanation is that the absence of the eCB-LTD mechanism permits LTP to occur. If CB1R concurrently mediate both LTP and LTD, the application of a CB1R antagonist could potentially clarify this situation. Indeed, there have been reports of eCB-dependent LTP in different brain aeras, resulting in an increase neurotransmitter release mediated by either homosynaptic mechanisms, or heterosynaptic processes involving CB1Rs located on astrocytes or GABAergic neurons (Navarrete and Araque, 2010; Piette et al., 2020). Lastly, it is worth noting that while eCBs may potentially enhance the induction of hippocampal LTP by dampening inhibitory transmission (as suggested by Carlson et al., 2002), this scenario can be ruled out in our current experiments that were conducted in the presence of a GABAAR antagonist.

Numerous studies have shown that LTP is affected in the hippocampus of Ts65Dn mice (Kleschevnikov et al., 2004; Costa and Grybko, 2005; Fernandez et al., 2007; Duchon et al., 2020), in the perirhinal cortex (Roncacé et al., 2017) and the striatum (Di Filippo et al., 2010), as well as in the hippocampus of other mouse models of DS; see for review (Bartesaghi, 2023). Most of these studies were done without blockade of inhibitory transmission. The deficit of LTP can be rescued by reducing the magnitude of GABA-mediated signaling through treatment with GABA_A_R antagonist (Kleschevnikov et al., 2004; Costa and Grybko, 2005; Fernandez et al., 2007; Duchon et al., 2020), or by reversing GABA_A_ R signaling, GABA being excitatory rather than inhibitory in Ts65Dn mice (Deidda et al., 2015). In our study, it is possible that the induction of LTP was facilitated by pharmacological inhibition of inhibitory transmission (Thomazeau et al., 2017). It would be compelling to replicate the experiment without the pharmacological blockage of inhibitory transmission to confirm our findings. However, a study conducted on the somatosensory cortex demonstrated that in Ts65Dn mice, the balance between excitatory and inhibitory transmission might remain mostly unaltered, which was indicated by a decrease in the frequency of both excitatory and inhibitory spontaneous synaptic activities (Cramer et al., 2015).

Importantly, the alteration in synaptic function observed in Ts65Dn mice could also be explained by non-neuronal changes, such as defects in astrocyte and/or microglia function. Indeed, while both have been shown to participate actively in synaptic transmission and plasticity mechanisms (Papouin et al., 2017; Marinelli et al., 2019; Stogsdill et al., 2023), new findings are challenging the neurocentric view of DS and providing new insights into the contribution of other brain cell types, such as astrocytes and microglia, to the pathophysiology of DS (Ponroy Bally and Murai, 2021; García and Flores-Aguilar, 2022).

### 4.3. Polygenic effects

How can we account for the differences in LTP observed between Ts65Dn and mBACtgDyrk1a mice? The partial Ts65Dn model provides a genetic environment more akin to that found in DS, with trisomy of over one hundred genes. However, the regulatory relationships between these genes are currently unknown, and overexpression of genes on chromosome 21 could lead to modified expression of genes on other chromosomes. While comparative analyses of gene expression on other chromosomes have demonstrated that gene deregulations are specific to Hsa21 in DS fetuses (Mao et al., 2003), research on the Ts65Dn model has shown that there is a global alteration in gene expression in these mice (Chrast et al., 2000; Lyle et al., 2004). Furthermore, gene products can interact with one another, creating compensatory or exacerbating mechanisms, which can affect synaptic parameters in Ts65Dn mice differently, reflecting the intricacy of the gene effects associated with this condition. In simpler terms, the deficits observed in Ts65Dn mice that are not present in mBACDyrk1a mice could be attributed to detrimental mechanisms arising from the overexpression of genes other than Dyrk1a. Conversely, the absence of defects in Ts65Dn that are seen in mBACDyrk1a mice could indicate the synergistic and/or compensatory activities of these three-copy genes. According to Dowjat et al. (2007), the amount of DYRK1A protein in the brains of Ts65Dn animals is 1.5 times higher. However, it is important to confirm whether this rate is consistent specifically within the PFC of the Ts65Dn animals. Currently, little is known about the spatiotemporal regulation of DYRK1A expression, or whether the overexpression of Dyrk1a and other trisomal genes in mouse models of DS varies across age, sex, and brain region throughout development (Stringer et al., 2017). Recent research has shown that the expression of DYRK1A protein in P15 mice is sex dependent in Ts65Dn mice, with significant overexpression in males but not in females (Hawley et al., 2022). This suggests that brain development in male and female Ts65Dn mice may have different trajectories, underscoring the significance of conducting future DS mouse model studies that include both males and females.

### 4.4. Limitations of the Ts65Dn mouse

It is important to note that the Ts65Dn animals have triplication of 50 non-orthologous Mmu17 genes in addition to Mmu16 orthologous genes, which may complicate the genotype/phenotype relationship and contribute to the observed synaptic phenotypes. To address this issue, future experiments could be performed on the refined Ts65Dn model recently developed, the Ts66Yah, which retains the major features of DS, but showed an overall milder phenotype than Ts65Dn mice (Duchon et al., 2022). However, it is worth noting that the partial Ts65Dn model does not overexpress the Hsa21 orthologous genes carried by Mmu10 and Mmu17, which raises questions about the relevance of this partial mouse model as a standard for DS research (Guedj et al., 2023). Therefore, it is important to conduct comparative studies in different mouse models for DS, each with its limitations in recapitulating the full spectrum of human DS. To fully compare the Ts65Dn and mBACtgDyrk1a mouse models, additional experiments such as examining basal glutamatergic transmission, mGluR3-LTD, dendrites and spines morphology would be necessary (see Thomazeau et al., 2014).

## 5. Conclusion

Overall, the parallel between this more complete Ts65Dn DS model and the monogenic Dyrk1a overexpression model highlights the complexity of the physiological and pathological mechanisms responsible for the neurocognitive symptoms of DS and underscores the importance of exploring new therapeutic strategies other than targeting DYRK1A activity.

## Data availability statement

The raw data supporting the conclusions of this article will be made available by the authors, without undue reservation.

## Ethics statement

The animal study was approved by Animals were treated in compliance with the European Communities Council Directive (86/609/EEC) and the United States National Institutes of Health Guide for the care and use of laboratory animals. The French Ethical committee authorized this project (APAFIS#3279–2015121715284829 v5). The study was conducted in accordance with the local legislation and institutional requirements.

## Author contributions

AT and OM designed the research. AT and OL carried out the experiments. AT analyzed the data. AT and OM wrote the manuscript. All authors contributed to the article and approved the submitted version.

## Funding

This work was supported by INSERM, CNRS, ANR “DsTher” (OM), Fondation Jérôme Lejeune (OM and AT) and Fondation pour la Recherche Médicale (OM and AT). The funders had no role in study design, data collection and interpretation, or the decision to submit the work for publication.

## Conflict of interest

The authors declare that the research was conducted in the absence of any commercial or financial relationships that could be construed as a potential conflict of interest.

## Publisher’s note

All claims expressed in this article are solely those of the authors and do not necessarily represent those of their affiliated organizations, or those of the publisher, the editors and the reviewers. Any product that may be evaluated in this article, or claim that may be made by its manufacturer, is not guaranteed or endorsed by the publisher.
